# Air Pollution Exposures During Adulthood and Risk of Endometriosis in the Nurses’ Health Study II

**DOI:** 10.1289/ehp.1306627

**Published:** 2013-11-13

**Authors:** Shruthi Mahalingaiah, Jaime E. Hart, Francine Laden, Ann Aschengrau, Stacey A. Missmer

**Affiliations:** 1 Department of Obstetrics and Gynecology, Boston University School of Medicine, Boston, Massachusetts, USA; 2 Channing Division of Network Medicine, Department of Medicine, Brigham and Women’s Hospital and Harvard Medical School, Boston, Massachusetts, USA; 3 Exposure, Epidemiology, and Risk Program, Department of Environmental Health, and; 4 Department of Epidemiology, Harvard School of Public Health, Boston, Massachusetts, USA; 5 Department of Epidemiology, Boston University School of Public Health, Boston, Massachusetts, USA; 6 Department of Obstetrics, Gynecology, and Reproductive Biology, Brigham and Women’s Hospital and Harvard Medical School, Boston, Massachusetts, USA

## Abstract

Background: Particulate matter and proximity to large roadways may promote disease mechanisms, including systemic inflammation, hormonal alteration, and vascular proliferation, that may contribute to the development and severity of endometriosis.

Objective: Our goal was to determine the association of air pollution exposures during adulthood, including distance to road, particulate matter < 2.5 μm, between 2.5 and 10 μm, and < 10 μm, (PM_2.5,_ PM_10–2.5_, PM_10_), and timing of exposure with risk of endometriosis in the Nurses’ Health Study II.

Methods: Proximity to major roadways and outdoor levels of PM_2.5,_ PM_10–2.5_, and PM_10_ were determined for all residential addresses from 1993 to 2007. Multivariable-adjusted time-varying Cox proportional hazard models were used to estimate the relation between these air pollution exposures and endometriosis risk.

Results: Among 84,060 women, 2,486 incident cases of surgically confirmed endometriosis were identified over 710,230 person-years of follow-up. There was no evidence of an association between endometriosis risk and distance to road or exposure to PM_2.5_, PM_10–2.5_, or PM_10_ averaged over follow-up or during the previous 2- or 4-year period.

Conclusions: Traffic and air pollution exposures during adulthood were not associated with incident endometriosis in this cohort of women.

Citation: Mahalingaiah S, Hart JE, Laden F, Aschengrau A, Missmer SA. 2014. Air pollution exposures during adulthood and risk of endometriosis in the Nurses’ Health Study II. Environ Health Perspect 122:58–64; http://dx.doi.org/10.1289/ehp.1306627

## Introduction

Endometriosis is defined as the presence of endometrial-like tissue outside of the lining of the uterus and may be either asymptomatic or accompanied by menstrual cycle pain and pelvic pain of varying severity. Surgical visualization is the gold standard for establishing a diagnosis of endometriosis. However, patients may be given a diagnosis based on clinically suspected disease based on history, physical examination, and imaging findings (Hsu et al. 2010). Endometriosis is staged based on the extent of surface area and locations involved from mild to severe disease. The reported prevalence of endometriosis varies by clinical population and method of ascertainment, from 4% to 50% (Abbas et al. 2012). The prevalence of undiagnosed endometriosis of all stages in the general population has been estimated at 11%, (Buck Louis et al. 2011). However, the likely prevalence of severe endometriosis is < 2% (Zondervan et al. 2002). In a study of white females, 15–49 years of age in Rochester, Minnesota, from 1970–1979, presenting to area hospitals and diagnosed with endometriosis, Houston et al. (1987) described the incidence of endometriosis by level of diagnostic certainty. There were 252 cases/100,000 person-years of endometriosis defined by the gold standard (histologically confirmed or surgically visualized) (Houston et al. 1987). In a previous study evaluating *in utero* exposures and incidence of endometriosis of the first 10 years of follow-up in the Nurses’ Health Study II (NHSII) cohort, the incidence of self-reported laparoscopically confirmed endometriosis was 298 cases/100,000 person-years among women 25–42 years of age (Missmer et al. 2004b).

Endometriosis is observed rarely in nonhuman primates, although animal models have been developed using transplantation of autologous endometrial tissue or human endometrial tissue into the peritoneum (Grummer 2006). In monkeys (*Macaca mulatta* or rhesus), endometriosis has been induced through long-term exposure to radiation (Fanton and Golden 1991; Splitter et al. 1972) and chronic exposure to dioxin compounds (Rier et al. 1993), with a minimum latency period of 7 years (Fanton and Golden 1991; Rier et al. 1993; Wood et al. 1983). There are limited data in humans regarding the etiologically relevant time window of exposure.

Several factors may contribute to endometriosis development and disease severity, including anatomic (Breech and Laufer 1999), anthropometric (Missmer et al. 2004a), hormonal, immunologic (Siristatidis et al. 2006), inflammatory, and genetic factors (Augoulea et al. 2012; Nyholt et al. 2012). Inflammatory markers are elevated in women with endometriosis (Gentilini et al. 2011); a state of inflammation is considered both to be a result of the disease and to perhaps promote disease progression (Agic et al. 2006).

Air pollution exposures include traffic-related exhaust (diesel and nondiesel) as well as particulate matter (PM) characterized by its size fraction. Smaller sizes of particulate matter, such as those ≤ 2.5 μm in diameter (PM_2.5_), can cross into the blood stream, deposit at distant tissues, and promote local and systemic inflammation (Brook et al. 2010; Calderón-Garcidueñas et al. 2011). Laboratory studies have demonstrated that diesel exhaust particles have hormonal activity in estrogenic and androgenic activity assays (Misaki et al. 2008; Oh et al. 2008; Sidlova et al. 2009; Wang et al. 2005). In a rat model of the *in utero* and postnatal effects of diesel exhaust on endometriosis, exposure to diesel exhaust enhanced persistence of endometriosis lesions, defined by persistence of surrounding collagen fibers (Umezawa et al. 2011). In the present study, we evaluated the association of exposure to traffic-related exhaust and PM during adulthood with the incidence of endometriosis in the NHSII cohort.

## Methods

**Figure 1:**
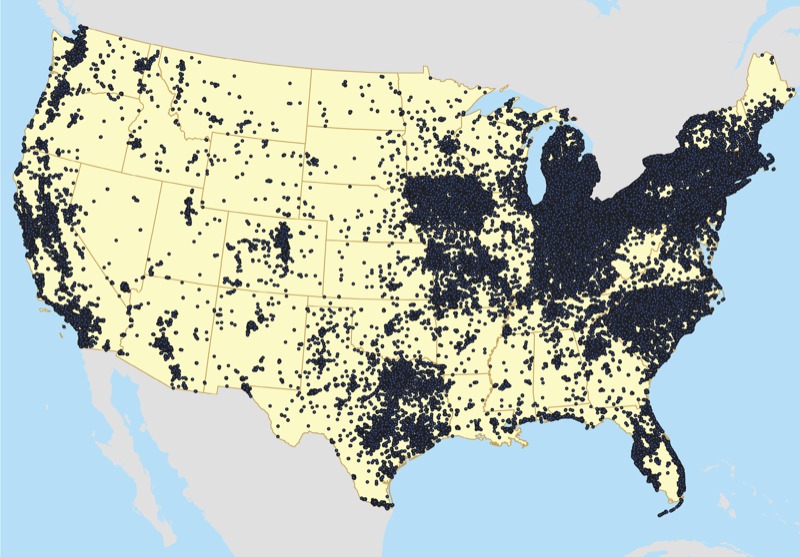
Geographic distribution of nurses’ addresses, 1989–2007.

*Study population*. Women in this study were participants in the NHSII, an ongoing prospective cohort study with a one-time enrollment in 1989 and 10 completed biennial follow-up surveys through May 2007. A total of 116,687 female registered nurses completed the baseline questionnaire. Nurses ranged in age from 25 to 43 years, and initially resided in 14 of the United States: California, Connecticut, Indiana, Iowa, Kentucky, Massachusetts, Michigan, Missouri, New York, North Carolina, Ohio, Pennsylvania, South Carolina, and Texas. However, as of the mid-1990s, they have resided in all 50 states and the District of Columbia (Figure 1). Questionnaires were used to collect information regarding the incidence of disease outcomes and a variety of biologic, environmental, dietary, and lifestyle risk factors. Surveys used to conduct this analysis were updated and mailed biennially, and had a > 90% response in each questionnaire cycle. Of the initial enrolled cohort, 93% were Caucasian—representative of the ethnic diversity of nurses in the United States at enrollment—and most resided in urban areas in the northeastern and midwestern United States. Women were included in the current study if they were alive, continued to respond to questionnaires throughout the course of follow-up, and did not have a diagnosis of endometriosis before 1993. For assessing exposure, the women also had to have at least one home address within the continental United States that could be geocoded to the street segment level. The study was approved by the institutional review boards of Boston University School of Medicine/Boston Medical Center and Brigham and Women’s Hospital. Informed consent was implied through the return of questionnaires.

*Case ascertainment*. In 1993, the participants were asked if they had “ever had physician-diagnosed endometriosis.” If they responded “yes,” they were asked to indicate whether the diagnosis was laparoscopically confirmed, because surgical diagnosis is the current diagnostic gold standard (Hsu et al. 2010). These questions were asked again on all subsequent biennial questionnaires. As described previously (Missmer et al. 2004a), a validation study was performed in 1994 based on a medical record review of a random sample of the 1,766 nurses who reported physician-diagnosed endometriosis after enrollment into the NHSII. The 131 women whose records were reviewed included 105 who reported a laparoscopic diagnosis of endometriosis. Among these women, medical record review confirmed 93 cases, and the other 12 received clinical care for endometriosis, despite having no visual confirmation of disease during laparoscopy. Of the 26 women who did not report laparoscopic diagnosis, records indicated a clinical diagnosis of endometriosis in 14, and no evidence of a diagnosis in the remaining 12 (Missmer et al. 2004a).

Women who reported endometriosis on the 1993 questionnaire were excluded from the present study. Women who reported a laparoscopic diagnosis of endometriosis after 1993 through 2007 were included as cases. Women who reported a clinical diagnosis of endometriosis without laparoscopy did not contribute any additional person-time but were allowed to re-enter the analysis in the future as a case if endometriosis was later confirmed by laparoscopy. The mid-point between the receipts of the questionnaire before and after diagnosis was assigned as the date of diagnosis.

*Exposure assessment*. Residential address information was updated in the NHSII cohort every 2 years as part of the questionnaire mailing process, and was geocoded to obtain latitude and longitude for each mailing address from 1989 through 2007. We used geographic information system (GIS) software (ArcGIS, version 9.2; ESRI, Redlands, CA) to calculate the distance to the nearest road (meters) at each residential address as a proxy measure of all exposures related to traffic. Road segments in the 2000 U.S. Census Topologically Integrated Geographic Encoding and Referencing system (TIGER) (U.S. Census Bureau 1992) files were selected by U.S. Census feature class code to include A1 (primary roads, typically interstate highways, with limited access, division between the opposing directions of traffic, and defined exits), A2 (primary major, non-interstate highways and major roads without access restrictions), or A3 (smaller, secondary roads, usually with more than two lanes) road segments. The shortest distance between each address and the closest road segment was calculated. Analyses were conducted using the distance to the closest of all three road types and distance to the two largest road types (A1, A2). Based on the distribution of distance to road in this cohort and previous exposure studies showing exponential decay in exposures with increasing distance from a road, for our primary analyses we categorized distance to road as 0–50, 50–200, or ≥ 200 m (Adar and Kaufman 2007; Karner et al. 2010; Lipfert et al. 2006; Lipfert and Wyzga 2008; Sahlodin et al. 2007; Zhu et al. 2002). We also considered cut-points up to 500 m in sensitivity analyses.

Predicted ambient exposure to particulate matter < 10 μm in aerodynamic diameter (PM_10_) and PM_2.5_ was available for each month since January 1988 through 2007 at the home address(es) of each cohort member. These values were generated from nationwide expansions of previously validated spatiotemporal models (Yanosky et al. 2008a, 2009). The models used monthly average PM_10_ and/​or PM_2.5_ data from the U.S. Environmental Protection Agency (EPA) Air Quality System, a nationwide network of continuous and filter-based monitors, as well as monitoring data from various other sources (U.S. EPA 2013). The models also used GIS to incorporate information on several geospatial predictors. All PM data and GIS data were used in generalized additive statistical models (Yanosky et al. 2008b) with smooth terms of space and time to create separate PM prediction surfaces for each month. Because monitoring data on PM_2.5_ are limited before 1999, PM_2.5,_ before 1999, was modeled using data on PM_10_ and airport visibility data (Yanosky et al. 2009). PM_2.5–10_ was estimated by subtracting monthly average values for PM_2.5_ from those for PM_10_. We calculated three different time-varying exposure measures: the average air pollution in the prior 2 calendar years, the average air pollution in the prior 4 calendar years, and the cumulative average exposure.

*Additional covariates*. We examined possible confounding by age (in months), race/ethnicity (African American, Asian, Caucasian, Hispanic, other), age at menarche, and—updated biennially—smoking status (current/former/never), body mass index (BMI), parity, oral contraception use, infertility (ever and current), ever performed rotating shift work (Schernhammer et al. 2011), region (Northeast, Midwest, West, South), and area-level socioeconomic status (SES)(census-tract level, median home value, and median family income). Variables selected for potential inclusion in the model included those identified in prior studies to correlate with endometriosis (parity, age at menarche) (Missmer et al. 2004a) or with exposure (elevation, land use) (Hart et al. 2009). Each potential confounder (or set of indicator variables) was added separately to a model that included age and race, *a priori*. We defined confounders as variables that changed the coefficient of the main effect of traffic exposure or PM by at least 10% when added to this basic model (Greenland 1989).

*Statistical analysis*. Time-varying Cox proportional hazards models were used to assess the relation of laparoscopically confirmed endometriosis with exposure to traffic or particulate matter. Person-time accrued from 1 September 1993 until first surgical diagnosis of endometriosis, hysterectomy, menopause, loss to follow-up, cancer diagnosis (other than non-melanoma skin cancer), date of death, or the end of follow-up, whichever occurred first. Person-time was calculated starting in 1993. This allowed for the calculation of up to 4 years of previous particulate exposure from baseline enrollment in 1989 and for the inclusion of prospective cases only. Person-time was excluded from follow-up for any period in which the home address was outside of the continental United States or was unable to be geocoded to the street segment level. Hazard ratios (HRs) and 95% CIs were calculated for categories of distance to road or for each 10-μg/m^3^ increase in PM. All Cox models were stratified by age in months and calendar year.

To determine whether associations of traffic exposure and air pollution with endometriosis differed by personal characteristics, we examined effect modification by parity (nulliparous or parous), overweight/obesity (BMI > 25 or ≤ 25 kg/m^2^), smoking status (ever or never smoker), age at menarche (< 12 vs. ≥ 12 years), infertility (current or ever versus never), and rotating shift work (ever vs. never). For each characteristic we performed time-varying stratified models (except for age at menarche) to obtain stratum-specific effect estimates and 95% CIs. To determine if there was statistically significant (*p* < 0.05) effect modification, we also ran models that included multiplicative interaction terms. To account for potential regional differences in traffic volume or PM composition, we also performed models stratified by region of the country.

The relation between endometriosis and infertility is complex. Of the women who both have endometriosis and are infertile, endometriosis may be either causative or incidental (Missmer et al. 2004a). Furthermore, women with infertility may undergo more diagnostic testing including laparoscopic evaluation that may identify endometriosis. Because there may be a diagnostic bias in women with infertility, to reduce this bias, we performed a sensitivity analysis restricted to those without infertility in two ways: *a*) reported at anytime during follow-up and *b*) ever versus never. Statistical analyses were performed in SAS version 9.2 (SAS Institute Inc., Cary, NC). An alpha level of 0.05 was used to define statistical significance.

## Results

A total of 84,060 women were in this study population for analyses of residential proximity to road, PM_10_, PM_10–2.5_, and PM_2.5_. The mean (± SD) age over the full course of follow-up was 42.0 ± 5.1 years, the cohort was mostly parous, and more than two-thirds were never-smokers. Age-standardized characteristics of the cohort for the full duration of follow-up are presented in Table 1. There was little difference in covariates among the distance to road categories, except for decreasing parity levels nearer to roadways and more women living nearer to roadways in the Northeast. The mean and median levels of each of the PM metrics were similar for each averaging period, and there were wide ranges in all pollutants (Table 2).

**Table 1:** Age-standardized characteristics^*a*^ of the cohort (*n* = 84,060) over the entire period of follow-up (1993–2007).

Characteristic	Entire cohort	By distance (m) to nearest A1–A3 roadway (m)^*b*^		
		≥ 200	51–199	0–50
Age (years)^*c*^	42.0 ± 5.1	42.1 ± 5.1	41.9 ± 5.2	42.0 ± 5.3
BMI (kg/m^2^)	25.9 ± 6.0	25.8 ± 5.9	26.0 ± 6.1	26.4 ± 6.4
Census tract median income ($10,000)	6.68 ± 2.38	6.75 ± 2.29	6.66 ± 2.47	6.33 ± 2.56
Census tract median home value ($100,000)	1.72 ± 1.24	1.67 ± 1.08	1.84 ± 1.41	1.76 ± 1.57
Caucasian race	94	94	93	93
Age at menarche (years)
< 12	23	23	23	23
12	30	30	30	31
> 12	46	47	46	46
Parity
Nulliparous	18	16	21	25
Parous	82	84	79	75
Oral contraception use
Never	14	13	16	15
Past	75	75	73	74
Current	10	10	10	10
Cigarette smoking
Never	67	68	66	65
Current	9	8	10	10
Former	24	24	24	24
Infertility
Yes	4	4	4	4
No	94	94	94	94
Ever performed rotating shift work
Yes	69	69	69	70
No	31	31	31	30
Region
Northeast	34	32	37	42
Midwest	34	35	31	30
West	15	14	20	15
South	17	19	13	13
Values are mean ± SD or percent. ^***a***^Values are standardized to the age distribution of the study population. ^***b***^Each cohort member may be in multiple ­distance categories over follow-up. ^***c***^Values are not age-adjusted				

**Table 2:** Distributions of the PM pollution metrics among 84,060 women in the Nurses’ Health Study II.

Metric	Mean ± SD	Median (IQR)	Minimum	Maximum
2-year average
PM_10_	23.2 ± 6.2	22.4 (6.9)	3.7	72.3
PM_10–25_	9.2 ± 4.6	8.3 (5.2)	0.0	54.0
PM_2.5_	14.0 ± 3.0	14.0 (4.1)	2.0	28.2
4-year average
PM_10_	21.7 ± 5.5	21.0 (6.2)	4.0	60.1
PM_10–25_	8.4 ± 4.2	7.4 (4.9)	0.1	46.3
PM_2.5_	13.3 ± 2.7	13.3 (3.7)	2.2	24.1
Cumulative average
PM_10_	26.2 ± 6.5	25.4 (6.9)	5.1	80.7
PM_10–25_	10.9 ± 4.9	9.8 (5.4)	1.7	60.7
PM_2.5_	15.4 ± 3.1	15.5 (4.2)	2.7	29.3

Over 710,230 person-years of follow-up, there were a total of 2,486 incident cases of laparoscopically confirmed endometriosis. In the basic models adjusted only for age and calendar time, living closer to a roadway than the referent, regardless of specific road type or cut-point categorization used, was generally associated with small nonstatistically significant elevations in the risk of endometriosis, compared with living farther from a roadway (Table 3). Age, parity, oral contraception use, age at menarche, region, and area-level SES were the variables that met our definition of confounding most consistently. In fully adjusted models, associations were slightly attenuated. In region specific analyses, nonsignificant positive associations were primarily evident in the Northeast. Particulate matter exposures were not positively associated with endometriosis in the population as a whole (Table 4). Models excluding women reporting infertility (including 1,578 incident cases over 565,243 person-years of follow-up) also demonstrated similar results (data not shown).

**Table 3:** Basic and fully adjusted HRs (95% CIs) of endometriosis risk by residential proximity to roadway, among 84,060 women in the Nurses’ Health Study II, in the whole country and by region.

Exposure	Whole country (2,486 cases)				Region [adjusted HR (95% CI)^*a*^]			
	Person-years	Cases	Basic HR (95% CI)^*b*^	Adjusted HR (95% CI)^*a*^	Northeast (761 cases)	Midwest (898 cases)	West (334 cases)	South (493 cases)
Distance to A1–A3 roads (m)
0–50	82,982	308	1.08 (0.95, 1.22)	1.04 (0.91, 1.17)	1.10 (0.89, 1.35)	0.98 (0.78, 1.22)	0.96 (0.66, 1.39)	1.06 (0.77, 1.46)
51–199	182,567	700	1.11 (1.01, 1.22)	1.09 (0.99, 1.19)	1.07 (0.90, 1.27)	1.07 (0.91, 1.25)	0.99 (0.78, 1.28)	1.28 (1.03, 1.59)
≥ 200	440,204	1,478	Reference	Reference	Reference	Reference	Reference	Reference
Distance to A1–A3 roads (m)
0–50	82,982	308	1.11 (0.97, 1.27)	1.07 (0.93, 1.23)	1.22 (0.96, 1.55)	1.00 (0.78, 1.27)	0.98 (0.63, 1.51)	0.98 (0.70, 1.37)
51–499	410,164	1,488	1.10 (1.00, 1.20)	1.09 (0.99, 1.19)	1.20 (1.00, 1.43)	1.07 (0.92, 1.24)	1.03 (0.77, 1.38)	0.99 (0.81, 1.20)
≥ 500	212,607	690	Reference	Reference	Reference	Reference	Reference	Reference
Distance to A1–A2 roads (m)
0–50	8,889	36	1.11 (0.79, 1.56)	1.03 (0.73, 1.44)	1.08 (0.63, 1.84)	0.74 (0.40, 1.40)	0.69 (0.16, 2.91)	1.68 (0.81, 3.48)
51–199	36,796	119	0.91 (0.75, 1.10)	0.86 (0.71, 1.04)	0.73 (0.53, 1.00)	0.79 (0.55, 1.13)	1.22 (0.77, 1.93)	1.23 (0.79, 1.91)
≥ 200	660,068	2,331	Reference	Reference	Reference	Reference	Reference	Reference
^***a***^Adjusted for age, calendar time, race, current BMI, smoking status, parity, oral contraceptive use, age at menarche, infertility, ever performed rotating shift work, and census tract–level median income and median home value and region (in unstratified models). ^***b***^Adjusted only for age and calendar time.								

**Table 4:** Basic and fully adjusted HRs (95% CIs) of endometriosis risk for each 10-μg/m^3^ increase in particulate matter, among 84,060 women in the Nurses’ Health Study II in the whole country and by region of residence.

Exposure averaging time	Whole country (2,486 cases)		Region [adjusted HR (95% CI)^*a*^]			
	Basic HR (95% CI)^*b*^	Multivariable adjusted HR (95% CI)^*a*^	Northeast (761 cases)	Midwest (898 cases)	West (334 cases)	South (493 cases)
2-year
PM_10_	0.98 (0.92, 1.05)	0.94 (0.87, 1.02)	1.01 (0.84, 1.21)	0.96 (0.82, 1.11)	0.91 (0.80, 1.03)	0.82 (0.61, 1.09)
PM_10–2.5_	0.98 (0.89, 1.07)	0.91 (0.81, 1.02)	0.88 (0.62, 1.24)	0.99 (0.81, 1.20)	0.86 (0.69, 1.05)	0.76 (0.54, 1.07)
PM_2.5_	0.98 (0.85, 1.12)	0.95 (0.83, 1.10)	1.18 (0.84, 1.65)	0.88 (0.67, 1.16)	0.85 (0.66, 1.11)	1.00 (0.72, 1.37)
4-year
PM_10_	0.97 (0.90, 1.05)	0.92 (0.84, 1.00)	0.98 (0.80, 1.20)	0.90 (0.76, 1.06)	0.92 (0.80, 1.06)	0.78 (0.55, 1.11)
PM_10–2.5_	0.97 (0.88, 1.07)	0.88 (0.77, 1.00)	0.88 (0.62, 1.24)	0.99 (0.81, 1.20)	0.86 (0.69, 1.05)	0.76 (0.54, 1.07)
PM_2.5_	0.97 (0.83, 1.13)	0.92 (0.79, 1.07)	1.14 (0.80, 1.63)	0.80 (0.59, 1.09)	0.86 (0.65, 1.13)	1.08 (0.75, 1.55)
Cumulative
PM_10_	0.97 (0.91, 1.04)	0.93 (0.87, 1.01)	1.02 (0.86, 1.21)	0.95 (0.82, 1.10)	0.91 (0.80, 1.02)	0.78 (0.61, 1.00)
PM_10–2.5_	0.98 (0.91, 1.07)	0.92 (0.82, 1.02)	0.90 (0.65, 1.24)	0.99 (0.82, 1.19)	0.89 (0.73, 1.08)	0.72 (0.52, 1.00)
PM_2.5_	0.93 (0.81, 1.05)	0.91 (0.80, 1.04)	1.19 (0.87, 1.62)	0.87 (0.68, 1.12)	0.79 (0.61, 1.02)	0.92 (0.69, 1.23)
^***a***^Adjusted for age, calendar time, race, current BMI, smoking status, parity, oral contraceptive use, age at menarche, infertility, ever performed rotating shift work, and census tract–level median income and median home value and region (in unstratified models). ^***b***^Adjusted only for age and calendar time.						

**Figure 2:**
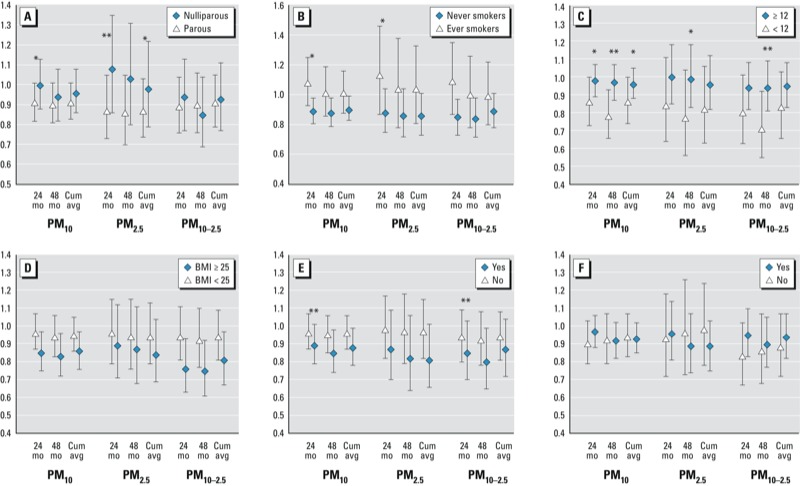
Examination of possible effect modification of a 10-μg/m^3^ increase in particulate matter and risk of incident endometriosis by parity (*A*), smoking status (*B*), age at menarche (*C*), BMI (*D*), infertility (*E*), and rotating shift work (*F*). All models are adjusted for age and calendar time, race, current BMI, smoking status, parity, oral contraceptive use, age at menarche, infertility, ever performed rotating shift work, region, and census tract–level median income and median home value as appropriate. *p for interaction < 0.10; **p for interaction < 0.05.

There was no evidence of effect modification in the distance to road models (data not shown). As shown in Figure 2, risks for a 10-μg/m^3^ increase in PM were elevated among the nulliparous, ever smokers, women with an age of menarche ≥ 12 years, women with a BMI ≤ 25, and women diagnosed with infertility. However, only a few interactions were statistically significant.

## Discussion

In this cohort of U.S. women, there was no statistically significant increased risk of endometriosis with exposures to traffic or ambient particulate matter (PM_2.5_, PM_10_, PM_10–2.5_) exposure during adulthood. To the best of our knowledge, this analysis represents the first human study to assess the relation between PM exposure and endometriosis.

Although many theories have been proposed, retrograde menstruation is the most widely accepted etiology for the initial establishment of peritoneal endometriosis (Sampson 1927). Because up to 85% of women have retrograde menstruation, it is further hypothesized that endometriosis occurs in susceptible women in the presence of abnormal endometrial cells (Flores et al. 2007; Halme et al. 1984; Kruitwagen et al. 1991). As described by Flores et al. (2007), viable endometrial cells deposited in the peritoneum adhere to peritoneal surfaces and invade under the stimulation of inflammatory cells and cytokines. Lesions then continue to infiltrate, develop a blood supply, and synthesize and secrete hormones (exact hormonal milieu depends on each lesion’s secretory profile) (Takeda et al. 2004). Systemic and local inflammation and oxidative stress (Andrade et al. 2010; Maybin et al. 2011) and hormonal alterations (including altered response to hormones and increased lesion hormone synthesis) are proposed mechanisms for endometriosis disease progression (Burney et al. 2007; Pan et al. 2007).

Air pollution has been shown to promote both local and systemic inflammation (*in vivo*) and increase oxidative stress (Brook et al. 2004, 2010), as well as possess hormonal activity through hormone receptor binding (*in vitro*) (Misaki et al. 2008; Oh et al. 2008; Sidlova et al. 2009; Wang et al. 2005). Thus, air pollution may engage several proposed pathways in endometriosis disease progression. In human studies and nonhuman primate animal models of endometriosis, there are elevations of proinflammatory cytokines in the peritoneal fluid in diseased subjects (Chen et al. 2010; D’Hooghe and Debrock 2002; D’Hooghe et al. 2001), as well as increased oxidative stress (Van Langendonckt et al. 2002). Additionally, dioxins (through the aryl hydrocarbon receptor) and endocrine disruptors found in diesel exhaust (including through the polycyclic aromatic hyrdrocarbon receptor) (Takeda et al. 2004) may influence the local hormonal milieu surrounding the lesion of endometriosis.

This study has a few limitations. We used ambient exposures at the residential address to estimate personal exposures. This could potentially lead to exposure misclassification in that we have no information on workplace exposures or the proportion of each day the woman spent at home, or on characteristics of the home (e.g., age, ventilation rate, air purification systems) that may influence the actual levels of ambient pollution exposure inside the home.

Restricting cases to those with laparoscopic confirmation may capture more “severe” cases only (Kennedy et al. 2005). However, among those with medical record review during our validation study (Missmer et al. 2004a), > 60% of these women were at stage I/II—minimal/mild disease at diagnosis. As mentioned previously, it is estimated that undiagnosed severe cases are < 2% of the general population (Zondervan et al. 2002). Although it is plausible that there are undiagnosed cases in the cohort, their prevalence among the > 70,000 comparison women is unlikely to explain our null results.

Although representative of the racial/ethnic and socioeconomic distribution of nurses at the time of cohort enrollment in 1989, the NHSII cohort is not representative of the general U.S. population. The NHSII cohort comprises predominantly Caucasian women living in neighborhoods of medium to high SES. Another limitation is that we are able to assess only associations with exposures during adulthood. Specifically, the women were 25–43 years of age at enrollment in 1989, and were 43–61 by the end of follow-up in 2007. This window of exposure may not be at the most etiologically relevant time window in relation to endometriosis disease pathogenesis. Hence, this investigation does not confer any insight into earlier-life exposure and endometriosis risk. As previously published from this cohort (Missmer et al. 2004b; Vitonis et al. 2010) and others (Kvaskoff et al. 2013; Treloar et al. 2010), exposures in earlier time windows, including intrauterine (diethylstilbestrol exposure, low birth weight) and childhood exposures (lean childhood body size, earlier age at menarche), have been associated with endometriosis. As has been suggested by Woodruff et al. (2008), it may be that exposures at these earlier and potentially more susceptible windows are the critical point of disease influence. Or it is possible that lifetime exposure information, including information on exposures during childhood, may be needed to elucidate the true relation between traffic and air pollution exposures and endometriosis.

This large nationwide prospective cohort of women also provided several important strengths. For this analysis, we had detailed biennially updated residential address history and included only residential addresses with a street segment–level geocoding match. Our exposure model provided spatially and temporally resolved predictions over the course of follow-up, which likely limits exposure misclassification compared with a single annual estimate or predictions from a central monitoring location. The geographic distribution represented by the participants of this study provided information on regions throughout the continental United States. Another strength was the prospective information on many potentially confounding covariates and effect modifiers.

Although our study did not support an association of adult exposures to PM with endometriosis risk, more research is needed on air pollution exposures and endometriosis.
